# High-Performance Structures of Biopolymer Gels Activated with Scleroprotein Crosslinkers

**DOI:** 10.3390/molecules30030627

**Published:** 2025-01-31

**Authors:** Miroslawa Prochon, Oleksandra Dzeikala, Szymon Szczepanik

**Affiliations:** Institute of Polymer and Dye Technology, Faculty of Chemistry, Lodz University of Technology, 90-537 Lodz, Poland; oleksandra.dzeikala@dokt.p.lodz.pl (O.D.); szymon.szczepanik@dokt.p.lodz.pl (S.S.)

**Keywords:** crosslinking, amino acid, cysteine, gelatin, scleroprotein films, biopolymer gels, scleroproteins, amino acid crosslinking, ion-redox initiators, keratin hydrolysate, Raman analysis, X-ray diffraction (XRD), SEM/EDS microscopy, ToF-SIMS spectrometry, gel permeation chromatography (GPC/SEC), biodegradable materials, disposable materials, polyvinyl alcohol (PVA)

## Abstract

The study explores innovative crosslinking processes for biopolymer gel materials using amino acids and ion-redox initiators to significantly enhance their structural and functional properties. Advanced analytical techniques, including FTIR, Raman spectroscopy, XRD, TEM, TGA, DSC, ToF-SIMS, SEM/EDS, GPC/SEC, and elemental analysis, were employed for comprehensive material characterization. The synthesized materials show potential applications in packaging and medicine, particularly for single-use products with short life cycles. Two crosslinking strategies were developed. The first combines gelatin with polyvinyl alcohol (PVA); keratin hydrolysate; and amino acids such as cysteine, hydroxyproline, proline, and histidine. The second employs endogenous cysteine, activated by ion-redox initiators, leveraging its trans-sulfuration ability to form highly stable polymer networks with optimized mechanical and thermal properties. Notably, the synergy between cysteine and potassium persulfate redox initiators proved particularly effective, making this approach attractive for industrial applications. This study introduces novel crosslinking methods and highlights the potential of amino acid-based strategies for designing advanced biopolymer gels with enhanced properties.

## 1. Introduction

Striving to obtain environmentally friendly materials with improved physical and chemical parameters is the essence of the current research on bioplastics in line with the principle of global sustainable economy. A material can be said to be biodegradable if it is decomposed into simple organic compounds, such as water, carbon dioxide, and biomass, without producing toxic products in all environments (salt water, fresh water, soil, and air) [1]. However, before a product undergoes decomposition, it must meet appropriate parameters during its use. Some materials are used as single-use products and end up in landfills, where they linger for many years—as is seen with plastics such as polyolefin bags and bottles. To prevent the generation of large deposits of waste, innovative solutions based on the use of biomass are under development [2,3,4]. Research on biodegradable materials of natural origin uses biopolymers rich in polysaccharide, polypeptide, or polynucleotide bonds. A biopolymer such as gelatin has great potential for application in the medical, cosmetic, food, agricultural, and other sectors. Thanks to its film-forming and membrane-forming properties, it is used for the production of soft or hard capsules in the manufacture of drugs [5,6]. Gelatin can be obtained from inexpensive sources such as waste or the by-products of production processes in the tanning, pharmaceutical, and food industries. Due to its biomimeticity and chemical composition, it is considered a renewable and biodegradable material. However, the poor mechanical properties of gelatin, especially under conditions of elevated moisture levels, limit its use in many other areas. To this end, the thermal stability and mechanical properties of collagen fiber-based films are being improved, for example, by introducing crosslinking agents, such as transglutaminase (TGase), an enzyme that occurs naturally in animal and plant tissues [7]. Thermostable proteins of casein, keratin, and soy protein isolate (SPI) affect the conformational changes and structural morphology of collagen. The crosslinking effect induced by TGase between the collagen fiber and proteins with higher thermostability promote the heat resistance and favorable mechanical properties of the film. Keratin, which is found in vertebrate skin, is responsible for these functions and exists in α-helix and β-folded sheet forms. These forms, in turn, are responsible for the enhanced activity of protein fibers based on crystalline structures embedded in an amorphous polypeptide matrix, which ensures the functionalization and stability of gelatin systems [8]. Scientists have already observed, in 2010 [6], the reinforcing effect of gelatin structures in the formation of covalent C-N bonds with phenolic compounds—caffeic acid (CA) or tannic acid (TA). The proposed pre-oxidation of phenolic structures in alkaline medium above pH = 9 contributed to the formation of quinone intermediate compounds capable of reacting with nucleophiles of reactive amino groups of the polypeptide chain. Such chemically active groups turned out to be the sulfhydryl group of cystine, the amide group of glutamines, the amine group of lysine, or the indole and imidazole rings of tryptophan and histidine [9]. The mobility of the insoluble hydrogels produced is variable and dependent on both the pH of the environment and the concentration of the oxidizing or reducing agents used in the crosslinking reaction. Lanlan Wei et al. [10] used keratin, which they modified using a reducing agent in the synthesis process. They modified the extracted keratin by reducing it with L-cysteine and applying curcumin as an antioxidant agent to the gelatin films. The morphology of the resulting films highlights the resulting structures and molecular interactions between curcumin, keratin and pectin, as identified by Fourier transform infrared spectroscopy and X-ray diffraction. The films have improved antimicrobial activity against strains of Staphylococcus aureus and Escherichia coli due to the addition of curcumin. These treatments are aimed at limiting the degradation progress of products such as foodstuffs due to oxidation processes, gas transport (O_2_ and CO_2_), water vapor, microbial activity, or color change in products such as meat products due to destructive effects of lipids.

The detailed mechanisms of amino acid crosslinking reactions in natural compounds require further exploration, opening perspectives for future research in this field. Therefore, the presented article deals with the development of a new concept of introducing scleroproteins, stabilizers, and crosslinking compounds into biopolymer gels, leading to a stable polymer network with improved mechanical and thermal parameters. For this purpose, the presence of sulfur -S, disulfide -S-S, thiol -S-H, as well as amide -C(O)-N and carbonyl -C=O groups was used to create a spatial network through the formation of stable interactions of covalent as well as ionic nature. The article addresses the challenge of our limited understanding of the crosslinking reaction mechanisms of amino acids in natural materials, which restricts their potential applications in biopolymer modification. The authors propose a novel approach that integrates scleroproteins, stabilizers, and crosslinking agents to create stable polymer networks with enhanced mechanical and thermal properties. Central to this concept is the utilization of reactive chemical groups, such as -S, -S-S, -S-H, -C(O)-N, and -C=O, to establish covalent and ionic bonds within the biopolymer gel structure.

## 2. Results and Discussion

### 2.1. Static-Mechanical Analysis

**Figure 1:**
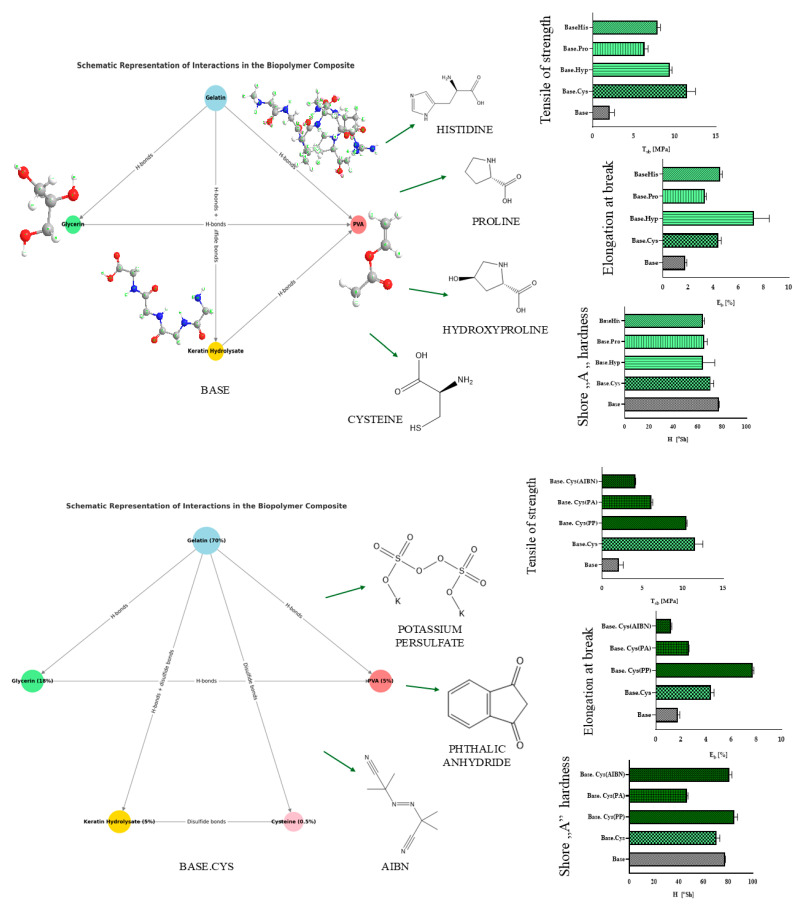
Shore A hardness; tensile strength; and elongation at a break of amino acid composites.

The use of keratin hydrolysate in combination with selected amino acids that acted as crosslinking agents was analyzed. The research included both single-component and hybrid systems, in which amino acids were introduced into gelatin–keratin matrices along with chemicals such as potassium persulfate (PP), phthalic anhydride (PA) and azobis(isobutyronitrile) (AIBN). The aim of the study was to increase the flexibility and deformation resistance of the materials by modifying their chemical structure. The results were analyzed in terms of key mechanical parameters, such as tensile strength (T_Sb_), elongation at break (E_b_), and Shore “A” hardness (H). The results showed a variety of mechanical properties depending on the type of amino acid used and the crosslinking agent chosen. The details are shown in Table 1, and the results are illustrated in the diagrams in Figure 1.

The use of two amino acids, cysteine and hydroxyproline, as crosslinking agents is particularly beneficial for gelatin matrices. The introduction of systems with keratin and hydroxyproline (Base.Hyp) and keratin and cysteine (Base.Cys) was aimed at creating a new structure in the spatial network of gelatin compositions. Trans-4-hydroxy-L-proline (hydroxyproline, C_5_H_9_NO_3_) contains active hydroxyl groups capable of reacting with reducing agents to form hydrogen bonds. This amino acid improves the mechanical properties because it is a key component of gelatin (about 10% in collagen) and presumably participates in the formation of interactions between gelatin and keratin. Similarly, cysteine ((R)-2-amino-3-mercaptopropionic acid, HSCH_2_CH(NH_2_)CO_2_H), plays an important role through its ability to form disulfide bridges and sulfide bonds that build a new structure for the gelatin–amino acid matrix.

The addition of cysteine present in the enzymatic hydrolysis of keratin, a key reinforcer of gelatin matrices, further enhances the stability of the system. Keratin has high stability due to the presence of numerous intermolecular bonds between polar and non-polar amino acids. In a further stage of the study, the selected initiator was used in combination with crosslinking agents, including cysteine, introducing it into the polymer matrices. Preliminary physicochemical studies have shown a similarity in the properties of the gel matrix to the samples with cysteine. The best mechanical parameters were distinguished by the Base.Cys(PP) material, in which the tensile strength increased compared to the native sample and approached the TS value of the single-component Base.Cys system. This is probably due to the presence of cysteine forming additional bonds with keratin in the gelatin matrix. The effectiveness of the resulting interactions increased after the addition of an initiator that activates the formation of ionic bonds in the polypeptide chain.

A significant role was played by the redox initiator, potassium persulfate (K_2_S_2_O_8_), which, as an oxidant, caused the oxidation of keratin, contributing to changes in its structure and chemical properties. This initiator activated sites on the gelatin and keratin in the polypeptide matrices much more effectively than the PA and AIBN initiators. The materials formed with their participation were more brittle and less flexible. The sulfhydryl group behaved similarly to the hydroxyl group but formed weaker hydrogen bonds due to the lower sulfur electronegativity. The reduction of ammonium to amine groups under the influence of (SO)_4_ groups was confirmed by spectroscopic, TOF-SIMS, and HPLC analyses. The oxidation phenomenon was observed in subsequent analyses, e.g., TOF SIMS at the molecular level, indicating the presence of new functional groups formed as a result of oxidation, such as sulfonic acid (-SO_3_H) or sulfonyl (-SO_2_-) groups, including the oxidation of disulfide bridges (S-S) to sulfonic acid (SO_3_). The infrared performed in FTIR showed the characteristic absorption bands associated with the presence of new oxidized groups, such as sulfonic acid (S=O, ~1030–1060 cm^−^^1^) or sulfonamide (N-S=O) groups. Changes in the elemental analysis in the atomic ratio of sulfur to other elements in the standard system versus systems with cysteine, proline, or histidine were observed.

Although AIBN generates a nitrile radical at temperatures above 60 °C, its action is more favorable in systems containing unsaturated bonds. In contrast, synthesis yields using PA were low due to the hydrolysis of this initiator to phthalic acid. The salts formed with the amino groups of proteins did not significantly improve the performance of gel matrices. This was demonstrated by both spectroscopic and TOF-SIMS analyses. Gels with the initiators AIBN ((CH_3_)_2_C(CN)N=NC(CH_3_)_2_CN) and PA (C_8_H_4_O_3_) were brittle, resulting in poorer strength parameters.

The hardness of the samples was tested using the Shore “A” scale method. The results showed that the use of cysteine as a crosslinking agent yields higher hardness values compared to other amino acids such as proline, hydroxyproline, and histidine. Of the initiators tested, PP and AIBN proved to be the most effective in increasing the hardness of the gel matrix, increasing it by nearly 7% compared to the reference sample (Base). The lowest hardness values were recorded for the PA-added sample, which had the lowest level of crosslinking. These data indicate that the introduction of PP and AIBN initiators into cysteine-crosslinked gel matrices favorably affects their hardness, while PA results in a softer and more flexible structure.

It is worth noting that only the samples showing notable improvements in mechanical properties were chosen for further analysis. Given the resource limitations, the study concentrated on these promising samples to conduct a thorough and impactful investigation of the most efficient crosslinking strategies.

### 2.2. FTIR and Raman Spectroscopy

**Figure 2:**
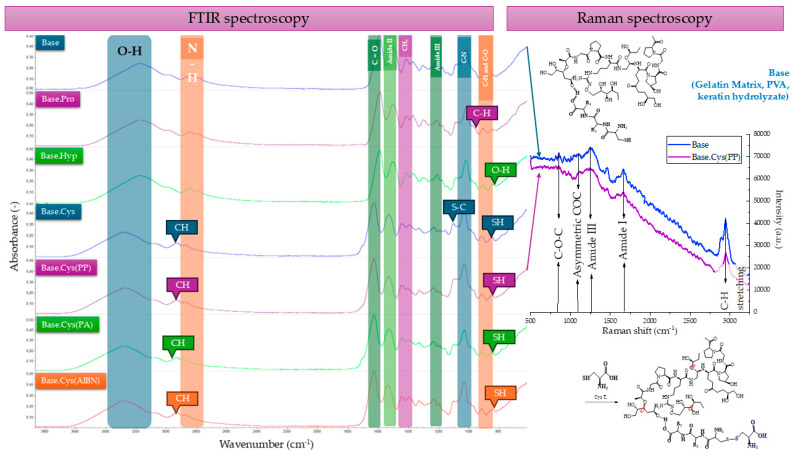
FTIR spectra of Base, Base.Pro, Base.Hyp, Base.Cys, Base.Cys(PP), Base.Cys(PA), and Base.Cys(AIBN), as well as IR Raman spectra of Base and Base.Cys(PP) used for comparative analysis.

The infrared spectroscopy analysis was aimed at studying the effect of the addition of fillers and amino acids on changes in the structure of the base material. The spectroscopic data of selected samples are shown in Figure 2, and the corresponding wavenumbers for the samples are presented in Table 2 and in Supplement S1 (Supplementary Materials) In the spectrum of the sample containing the redox initiator and cysteine, the presence of hydroxyl groups and a change in the intensity of their band in the range of 3000–3500 cm^−^^1^ were observed, as well as amine bands and carbonyl groups (CO). Signals characteristic of ester bonds at a wave number of 1700 cm^−^^1^ were also identified, as well as the following amine bonds: primary at 1650 cm^−^^1^, secondary at 1550 cm^−^^1^, and tertiary at 1200 cm^−^^1^.

In the case of a sample spectrum containing casein, a redox initiator, and cysteine, signals indicating the presence of covalent bonds resulting from the disulfide and monosulfide linkages of cysteine were detected at the intensity change in the 400–700 cm^−^^1^ range. This observation is further confirmed by the TOF-SIMS analysis, which identified a 120.02-unit quasimolecular ion consisting of a molecule and a weakly bound positive ion (CSH^+^). This fragment likely originates from a cysteine structure interacting via S-C, S-S, or SH covalent bonds. Additionally, the following signals related to amide bonds were noted: tertiary (1200 cm^−^^1^), secondary (1550 cm^−^^1^), and primary (1650 cm^−^^1^). Furthermore, a change in the intensity of interactions originating from hydroxyl groups was identified in the range of 3000–3500 cm^−^^1^. These findings collectively suggest that cysteine’s incorporation into the system contributes to structural features such as covalent bond formations, which is consistent with its known chemical behavior under oxidative conditions and supports matrix stabilization [11].

The spectrophotometric analysis confirmed the possibility of forming new connections between the gelatin matrix and the protein components as a result of the crosslinking process using the introduced amino acid agents. These results testify to the effective modification of the structure of the compositions via the chemical interactions between the components.

The FTIR spectroscopic analysis showed noticeable shifts in the ranges of functional groups on the Raman spectra. C-H stretching vibrations characteristic of organic compounds, especially carbon atoms connected by hydrogen bonds, are visible in both FTIR and Raman, but the intensity of the bands depends on the dipole activity (FTIR) or polarizability (Raman). The disappearance of the band at 2080 cm^−^^1^, corresponding to the N-H stretching vibrations of the -NH_3_^+^ group, originating from L-cysteine or the vibrations of triple bonds, e.g., C≡N resulting from the action of an oxidant, indicates changes in the structure of this amino acid. Similarly, the disappearance of N-H bands from secondary amide bonds (1550–1580 cm^−^^1^) suggests changes in the protein structure or peptide-related processes.

Raman signals can be intense due to the large change in polarizability in sulfur bond vibrations, which are in the range of 500–400 cm^−^^1^ for FTIR, indicating the involvement of thiol groups in proteins and amino acids, e.g., cysteine. The correlation of the FTIR results with Raman spectroscopy confirmed that the disappearance of the bands at 2080 cm^−^^1^ and the bands in the range of 1550–1580 cm^−^^1^ reflects changes in amide bonds and the breakdown of bonds associated with L-cysteine [12,13].

New signals appeared at 1250 cm^−^^1^, which corresponds to vibrations characteristic of peptides, indicating changes in their structural composition. In addition, asymmetric vibrations at 1121 cm^−^^1^ and 850 cm^−^^1^ suggest modifications related to proteins and peptides. Specifically, vibrations at 1121 cm^−^^1^ may originate from phosphorus groups, ether groups, S=O bonds, or systems with C-O-C bonds, reflecting alterations in these chemical structures. Similarly, vibrations at 850 cm^−^^1^ may be attributed to aromatic rings, C-S bonds, or cyclic ring structures, which are associated with the modification of biopolymers. The signals are more intense for groups that cause changes in polarizability, such as aromatic rings or systems with delocalized electrons, as observed in the Raman analysis. In the FTIR analysis, signals in these ranges are often more pronounced for groups that induce changes in dipole moment [14,15,16].

### 2.3. Analysis of the Contact Angle and Surface Free Energy

The tests conducted using the goniometer involved matrices crosslinked with the following amino acids: cysteine (HSCH_2_CH(NH_2_)CO_2_H), hydroxyproline (C_5_H_9_NO_3_), proline (C_5_H_9_NO_2_), and histidine (C_6_H_9_N_3_O_2_), as well as hybrid systems involving amino acid–initiator combinations (e.g., cysteine-AIBN, cysteine-phthalic acid anhydride, cysteine-K_2_S_2_O_8_). Due to the presence of keratin enzymatic hydrolysate in all matrices, cysteine, which is combined with initiators in the solvent casting synthesis process, was chosen as the amino acid for crosslinking in the second method (Section 3).

Measuring the contact angles allowed us to determine the nature of the surface of the tested gel films. In the case of distilled water, all compositions with amino acid-crosslinked matrices showed a contact angle above 90°, which means that the tested surface is hydrophobic (90° < Θ < 180°); conversely, in the case discussed below, amino acids and crosslinking initiators were used simultaneously, for which the angles were below 90°. Their hydrophobic character may be due to the presence of polar groups directed into the material structure. For ethylene glycol and diiodomethane, there was a significant decrease in the contact angle—in some cases it was reduced by up to half, indicating good wetting of the surface by these liquids (Θ < 90°). The highest contact angle values for glycol and diiodomethane were recorded in samples crosslinked with hydroxyproline (Hyp) and proline (Pro).

The addition of amino acids changes the nature of the surface from hydrophilic to hydrophobic. The polar component of the surface energy accounts for a small portion of the total energy, while the dispersive component has significantly higher values. Base.Cys and Base.Pro materials had the highest surface energy values, indicating they allow for a higher degree of crosslinking. The introduction of polar amino acids increases the proportion of the polar phase in the material, which is consistent with the presence of hydrogen bonds formed by macromolecules of gelatin, collagen, and keratin [17].

This type of polymer contains strongly polar carbon–nitrogen bonds which, due to differences in the electronegativity of groups such as -NH- or -(C=O)-, can form strong intermolecular interactions in the form of hydrogen bonds. At the same time, non-polar fragments, made up of repeating -CH_2_- groups, present in the main chain or side chains, give the polymer matrices a non-polar and hydrophobic character. This phenomenon is due to the minimal dipole moments between the carbon and hydrogen atoms, which cancel each other out due to their symmetrical arrangement [17,18].

The earlier addition of one of the three initiators during the synthesis of gel systems—azobis(isobutyronitrile) (AIBN, (CH_3_)_2_C(CN)N=NC(CH_3_)_2_CN), phthalic anhydride (PA, C_8_H_4_O_3_), or potassium persulfate (PP, K_2_S_2_O_8_)—before introducing the amino acid L-cesteine, influenced the changes in the values of the polar and dispersion components as well as the surface free energy of the SFE (Table 3).

In the compositions containing cysteine together with the initiator, a marked decrease in contact angle values was observed, which ranged from 42 to 62° compared to the native Base sample (106°). The system with the most polar character was Base.Cys(PP). The Base.Cys(PP) system shows a more polar character, which suggests partial hydrophilicity resulting from the polar–covalent interactions present in the material. Thus, while some compositions show hydrophobicity, others, especially those containing initiators, become more wettable and less hydrophobic. The contact angles for the other liquids, both inert and non-polar, were similar to those of the native Base sample. Nevertheless, the wettability of these samples was not optimal, which was reflected in other physicochemical parameters such as water adsorption capacity, surface chemical composition, protein binding capacity, solubility and chemical stability, and mechanical and thermal properties. The addition of initiators to the gelatin systems did not favorably improve the surface structure, as evidenced by an average 25% decrease in surface free energy (SEP) values. The initiators contributed to an increase in the dispersivity parameters and a decrease in the polarity of the systems.

A significant proportion of the polar phase has been reported in keratin compositions, which is confirmed by the mechanical test results. Less polar materials are more hydrophobic, and their components form a more durable structure, resulting in greater strength. In contrast, amino acid-containing compositions, characterized by a higher proportion of the polar phase, are more flexible but potentially more susceptible to decomposition and water vapor, which can lead to faster degradation, for example, in the environment.

Contact angle studies showed correlations with the results of the IR Raman analysis, where interactions were also identified at bands associated with C-O, C=O, C-H, O-H, and C-N groups. Correlations could also be seen in elemental analysis, which made it possible to identify the elements present in the samples. Materials crosslinked only with amino acids show greater hydrophobicity. The introduction of initiators leads to the formation of more polar interactions, which makes the system less stable and weakens its mechanical properties.

### 2.4. Differential Scanning Calorimetry (DSC) and Thermogravimetric Analysis (TGA)

**Figure 3:**
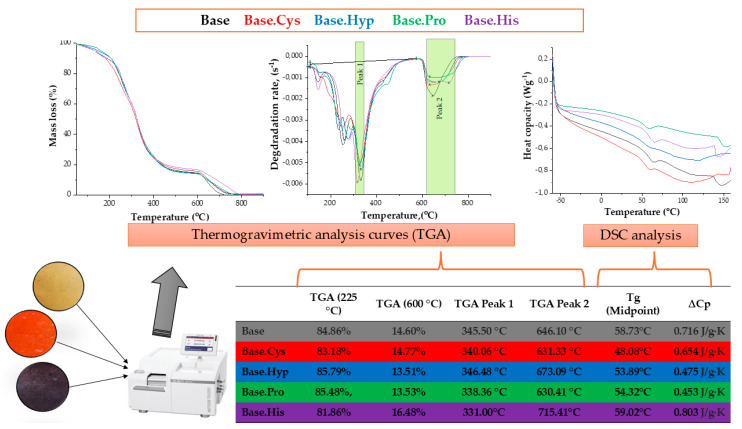
Thermogravimetric analysis curves (TGA) and comparison of DSC analysis curves for Base, Base.Cys, Base.Hyp, Base.Pro, and Base.His.

Thermogravimetric (TGA) studies made it possible to evaluate the behavior of gelatin compositions under changing temperature, which made it possible to determine their thermal stability. Softening temperature studies using the Vicat method showed an increasing trend due to amino acids crosslinking gelatin matrices. Based on these results, thermal analysis was carried out, showing the endothermic transformations occurring in the samples during heating under specific conditions. The results of the TGA and DSC analysis for selected materials are shown in Figure 3.

Using DSC (differential scanning calorimetry) analysis, the glassy and heliac transitions were determined, indicating interactions between gelatin and amino acids through hydrogen bond formation. On the other hand, TGA made it possible to estimate parameters related to mass loss during thermal processes.

The thermogravimetric curves (Figure 3) show a typical TGA thermogram for gelatin films [19]. Weight loss was analyzed as a function of temperature from room temperature to 900 °C. The graphs show that gelatin decomposes in two stages [20]. The first stage of decomposition occurs at 180–250 °C, where a weight loss of 13–16% was recorded, which can be attributed to the evaporation of the solvent, moisture, and water vapor. The second stage occurs in the range of 500–700 °C, with a mass loss of 81–85%, which is associated with nitrogen loss and the thermal degradation of the gelatin molecules [21,22,23].

DTG (thermogravimetric derivative) curves for gelatin and amino acid-crosslinked gelatin materials indicate the presence of four characteristic endothermic peaks. The first peak is observed in the range of 100–500 °C, and at a temperature of about 140–150 °C, water evaporation from the polymer network structure occurs. The next peak appears in the range of 230–250 °C, which corresponds to the beginning of the decomposition of the polyvinyl alcohol (PVA) contained in the matrix. At around 290 °C, the decomposition of glycerol begins, while in the 310–330 °C range, the decomposition of gelatin and the introduced amino acids, such as cysteine (Cys), hydroxyproline (Hyp), proline (Pro) and histidine (His), as well as their protein counterparts, occurs.

The last endothermic transformation occurs above 650 °C and is associated with the pyrolysis of the residual substances in the sample. These results indicate the complex nature of the thermal processes in gelatin materials and the significant influence of the amino acids used on their thermal stability and behavior at elevated temperatures.

In order to study the effect of additives on the thermal properties of the base composition containing PVA, keratin hydrolysate, and amino acids, DSC analysis was carried out. The study made it possible to determine the glass transition temperature and heat capacity (Figure 3).

Heating rate curves as a function of temperature (Figure 3) indicate the time-dependent thermal relaxation of gelatin-based polymers. These transitions probably reflect the physical behavior of gelatin and are consistent with data from the literature [20,24]. The study considered the effect of amino acids such as cysteine (Cys), hydroxyproline (Hyp), proline (Pro), and histidine (His) on the base material (Base). The first changes in heat flow, as seen in the DSC curves, are attributed to the transformation from a rigid and elastic state to a so-called “rubbery” state—viscoelastic state, which is the result of the Brownian motion of the molecules and macromolecules of the biopolymer [19].

After the glass transition, a distinct endothermic peak was observed, which can be attributed to the melting of the triple crystal helix structure of the polypeptides. This transition is associated with the dissociation and melting of ordered regions at the following softening temperatures: TmCys ≈ 48.08 °C, TmHyp ≈ 53.89 °C, TmPro ≈ 54.32 °C and TmHis ≈ 59.02 °C. During these transformations, at the level of chemical processes and synthesis, there are conformational changes in protein structures and reorganization of helices into so-called disorderly clusters, which limit the rotation of polypeptide side chains. An example of such a phenomenon is the physically bound water in keratin, which affects the nature of this protein [25]. In the DSC plots, the endothermic peak (Figure 3) results from the overlap of processes such as the evaporation of water, the melting and recrystallization of the fine crystalline forms of gelatin, as well as the association of the glass transition of α-amino acid blocks in the polypeptide chain.

Another endothermic peak occurs at about 150°C and is almost invisible for BaseHis and small for BaseCys. This indicates the greater thermal stability of these two materials, which is confirmed by the results of the mechanical tests (Section 3.3.1).

At temperatures above 200 °C, the decomposition of gelatin and cyclic amino acids such as proline, hydroxyproline, histidine, and cysteine are observed, as described earlier in the TGA studies. The transition from the glassy to viscoelastic phase can lead to the re-formation of helical gelatin structures and their partial aggregation in the denaturation process. With the introduction of pure amino acids into the system as crosslinking agents, it is possible to roll up the structures to their original conformation.

Comparing the thermal properties of different compositions, it can be seen that the highest decomposition initiation temperature occurs in the system with histidine (Figure 3). This material also requires more heat (∆Cp = 0.803 J/gK) compared to the other systems tested. A similar pattern was observed for BaseCys, which is consistent with previous TGA results and XRD analysis.

### 2.5. HPLC Analysis

**Figure 4:**
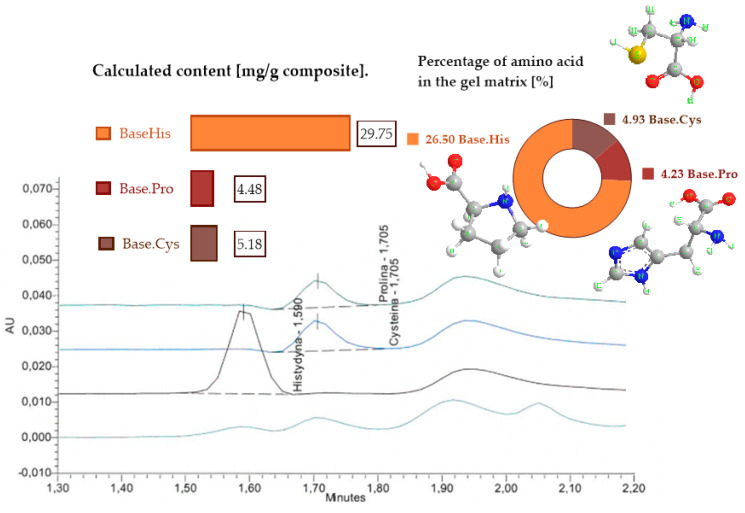
Calculated content and percentage of amino acids in gelatin matrices and HPLC chromatograms of the following amino acids: proline, cysteine, histidine.

The analysis of the content of histidine, cysteine, and proline in the polymer materials was carried out using reversed-phase liquid chromatography with UV detection (Figure 4). The figure shows the percentage of each amino acid in the materials analyzed. The HPLC chromatograms list the amino acids that are the crosslinking agents in the gelatin materials and determine their proportion in the gelatin matrices.

During the synthesis of gelatin materials by the solvent casting method, amino acids were introduced in an amount of 0.27% relative to the total weight of the components. The results of HPLC analysis showed that the content of amino acids in the finished compositions was much higher, which indicates their active presence in the base matrix. The matrix was gelatin (collagen), naturally containing histidine and proline, and keratin hydrolysate, which additionally provides cysteine, histidine, and proline.

According to the literature [26,27], cysteine is the predominant amino acid in fibrillar proteins, accounting for about 12–14% in them, and in keratin hydrolysates, its content averages 5.6% [26,27]. The HPLC analysis (Figure 4) showed that the cysteine content in gelatin compositions reaches a value of about 5.2%, which is the result of the introduction of enzymatic keratin hydrolysate into the matrix. Keratin hydrolysate contains fragmented short peptide chains, formed during enzymatic processing carried out at pH 6.5–8 and at temperatures below 100 °C.

The hydrolysis process breaks down telopeptide crosslinking chains, interrupting key covalent bonds. As a result, the resulting hydrolysate has active sites capable of binding additional crosslinking amino acids, such as cysteine (in an amount of 0.25%), which affects the secondary structure of the protein and reduces the length of the peptide chains. The remaining amount of cysteine (4.93%, as indicated in Figure 4) is mainly derived from keratin hydrolysate, and small amounts may originate from collagen [26,27].

The presence of proline and hydroxyproline, characteristic of collagen, introduces cyclic amino acids into the system, which form ordered and crystalline regions separated by groupings of neutral amino acids [28]. This structural organization significantly affects the physical properties of the materials, highlighting the key role of amino acids in shaping gelatin–amino acid systems.

The introduction of histidine as a crosslinking agent, as well as the use of cysteine and proline, significantly improved the relative elongation parameter (Eb), as discussed in the section on the static-mechanical analysis. This influence is closely related to the presence of hydrogen bonds, which, unlike the stronger covalent bonds with an energy of 30–120 kcal/mol, have lower geometrical and physical requirements, with energies in the range of 2–40 kcal/mol.

In the context of proteins and water, crystallographic studies show that hydrogen bond lengths average between 2.7 and 3.3 Å, with 3.0 Å being the most commonly observed value. An analysis of the matrices studied showed that hydrogen bonds in polymer networks are formed with the participation of water, which can act as both a proton donor and acceptor. Thus, proteins, being ampholytes, can orient themselves positively or negatively, which is due to their electrolytic nature [29,30,31,32]

In addition, low-energy barrier hydrogen bonds, which are more flexible in biological systems, play an important role in the structure and function of the network. [32,33].

The use of histidine, cysteine, and proline in the crosslinking of gelatin–amino acid matrices also provide valuable information about the orientation of protein side chains, which can often be ambiguous. An analysis of the surface wetting angles of these matrices, coupled with surface free energy (SEP) determinations, allows for a better understanding of the characteristics of the resulting surfaces and their interactions in aqueous environments, which may have important implications for the further design of such materials.

### 2.6. Elementary Analysis

**Figure 5:**
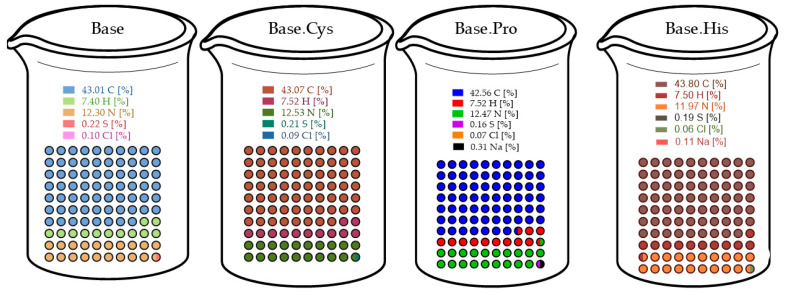
Comparison of elemental content in Base, Base.Cys, Base.Pro, and Base.His samples obtained by elemental analysis.

The elemental analysis of the studied samples allows for the detailed evaluation of the content of the main elements (C, H, N, S, Cl, Na) and their changes due to the modification of the biopolymer matrix by the addition of cysteine, proline, and histidine are shown in Figure 5. The results provide important information on the influence of individual amino acids on the chemical composition of the matrix, as well as identify potential mechanisms of crosslinking and chemical interactions in the studied systems.

In terms of carbon content (C), the Base sample has a value of 43.07 ± 0.05%, which is typical of collagen-based protein matrices. In the Base.Cys sample, the carbon content dropped slightly to 43.01 ± 0.01%. This may be due to the introduction of cysteine, whose molecules contain fewer carbon atoms compared to other matrix components. The Base.Pro sample, on the other hand, showed a further decrease in carbon content (42.56 ± 0.03%), which could be attributed to the specific structure of the pyrrolidine ring in proline, affecting the overall carbon ratio of the material. The Base.His sample stands out as having the highest carbon content (43.80 ± 0.08%), consistent with the presence of an imidazole ring in histidine, which is rich in carbon.

Hydrogen (H) content remains relatively stable in all samples, at around 7.5%. The slight decrease in the Base.Cys sample (7.40 ± 0.02%) may be due to the presence of thiol groups in cysteine, which do not provide additional hydrogen atoms compared to other amino acids. In contrast, the lack of major differences in the other samples suggests that the introduction of proline and histidine does not significantly affect the overall hydrogen content of the matrix.

The nitrogen (N) content is 12.53 ± 0.02% in the Base sample, which is typical for collagen-based protein matrices. The introduction of cysteine leads to a slight decrease in nitrogen content to 12.30 ± 0.01%. This is due to the lower number of amino groups in the cysteine molecule compared to the other amino acids in the matrix. Proline in the Base.Pro sample maintains a nitrogen content similar to that of the base sample (12.47 ± 0.06%), indicating that there are no significant chemical changes associated with the addition of this amino acid. In the case of histidine, the Base.His sample shows a significant decrease in nitrogen content to 11.97 ± 0.09%. This is consistent with the presence of the imidazole ring, which contains fewer amino groups relative to the molecular weight.

The sulfur (S) content in the Base sample is 0.21 ± 0.07% and does not change significantly in the Base.Cys sample (0.22 ± 0.07%). The minimal increase in sulfur content indicates the presence of thiol groups of cysteine, which are crucial for the formation of disulfide bridges that stabilize the matrix. In the Base.Pro sample, the sulfur content drops to 0.16 ± 0.05%, consistent with the absence of sulfur groups in proline. In the Base.His sample, the sulfur content remains at 0.19 ± 0.06%, indicating that histidine has no effect on this parameter.

The chlorine (Cl) content in the Base sample is 0.09 ± 0.01% and is likely related to the presence of collagen hydrolysate, which may contain chloride residues from the collagen extraction or processing process.

Sodium (Na) was detected only in the Base.Pro sample (0.31 ± 0.08%) and in a small amount in the Base.His sample (0.11 ± 0.04%). The presence of sodium in the Base.Pro sample may be due to residual salts used in the synthesis or processing of the amino acids.

The introduction of various amino acids into the gelatin matrix influences the elemental composition of the samples, confirming their involvement in chemical and structural modification. The increased sulfur content in the Base.Cys sample clearly indicates the participation of cysteine in the formation of disulfide bridges, which are crucial for the mechanical and chemical stability of the matrix. The decrease in nitrogen content in the Base.His sample confirms the influence of the imidazole ring of histidine, while the stable nitrogen content in the Base.Pro sample suggests the more neutral nature of proline in interactions with the matrix.

The results of the elemental analysis indicate significant differences in the chemical composition of the samples exist as a result of the introduction of different amino acids. Cysteine increases sulfur content and matrix stability through disulfide bridges, while histidine and proline modify the matrix to a lesser extent. The chlorine present in the samples is derived from collagen hydrolysate, confirming the chemical nature of the matrix. The elemental analysis of biopolymer gels showed that the addition of cysteine increased the sulfur content, suggesting the formation of disulfide bridges and the stabilization of the matrix. Histidine decreased the nitrogen content and increased the carbon content, reflecting the specific properties of the imidazole ring, while proline had less impact on the modification of the matrix. The results indicate that the different amino acids caused significant changes in the chemical composition and structure of the biopolymers, with a dominant role of cysteine in stabilizing the systems.

### 2.7. Structural Characterization of Samples: Amorphousness and Crystallinity Analysis Using Vicat Temperature and XRD Analysis

**Figure 6:**
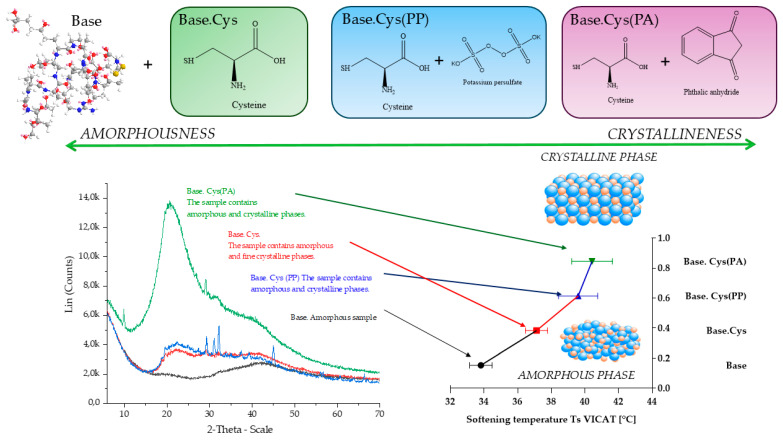
Effect of cysteine and initiators on the crystalline and amorphous structure of gelatinous materials. XRD and softening temperature (Vicat) analysis for Base, Base.Cys, Base.Cys(PA), and Base.Cys(PP).

Tests on gelatinous materials were carried out using the Vicat method to determine the softening temperature, which is a key parameter in assessing the thermal properties of materials of this type. The results shown in the Figure 6 confirm the amorphous nature of the tested materials, which do not have a clearly defined melting point. The softening temperature, obtained as an average of three measurements for each sample, increased by 14% on average in samples enriched with amino acids, crosslinking additives and initiators compared to the base matrix. This increase indicates the stabilization of the structure through stronger covalent and ionic interactions.

Amorphous materials, such as the gelatin materials studied, are characterized by a tangled arrangement of macromolecules resembling a cluster, resulting in low energy interactions (Figure 6). The introduction of amino acids initiates the formation of ordered segments, which can lead to partial crystallization, especially under conditions favorable for processing. These effects were further confirmed by X-ray diffraction (XRD) analysis, which showed the presence of finely crystalline peaks in the amino acid-containing structures compared to the initial sample with a typically amorphous structure [34].

The XRD studies were conducted for gelatin films crosslinked with cysteine and cysteine in the presence of the initiators potassium persulfate (PP) and phthalic acid anhydride (PA). The diffractograms showed significant differences in the position of the peaks characteristic of gelatin in samples enriched with cysteine and initiators compared to the base sample. Visible broad peaks in the range 2θ = 18°−27° for Base.Cys, Base.Cys(PP), and Base.Cys(PA) samples indicate a partial crystalline nature and confirm the reconstitution of the collagen-like triple helix [35,36]. The process of collagen microfilament denaturation assisted by the presence of cysteine and the initiators (PP and PA) was made visible in the appearance of new peaks with a distinct shift compared to the base sample [19].

The Base.Cys sample shows two distinct peaks at 2θ = 29.3° (0.157 nm) and 2θ = 41.8° (0.133 nm), which indicate local structural ordering. The addition of the PP initiator increases the number of peaks, with significant maxima at 2θ = 29.2° (0.158 nm), 2θ = 31.1° (0.149 nm), 2θ = 37.6° (0.126 nm), and 2θ = 66.3° (0.084 nm). The use of the PA initiator, in turn, generates a unique high-intensity peak at 2θ = 9.9° (0.418 nm), which is characteristic of the collagen triple helix. The emerging new peaks suggest the formation of a more organized crystal structure through the interactions of cysteine with the initiators and the gelatin matrix.

The presence of cysteine as a polar amino acid and the use of PP and PA initiators contribute to the formation of additional hydrogen bonds and crosslinking covalent bonds that strengthen the structure.

The XRD analysis confirms that the presence of fine crystalline regions correlates with the results of Vicat’s method and the DSC analysis, where higher glass transition temperatures and the increased mechanical hardness of materials enriched with cysteine and initiators were observed. The higher proportion of the crystalline phase translates into the higher thermal and mechanical stability of the systems, making these materials more resistant to degradation under service conditions.

### 2.8. SEM and TEM

**Figure 7:**
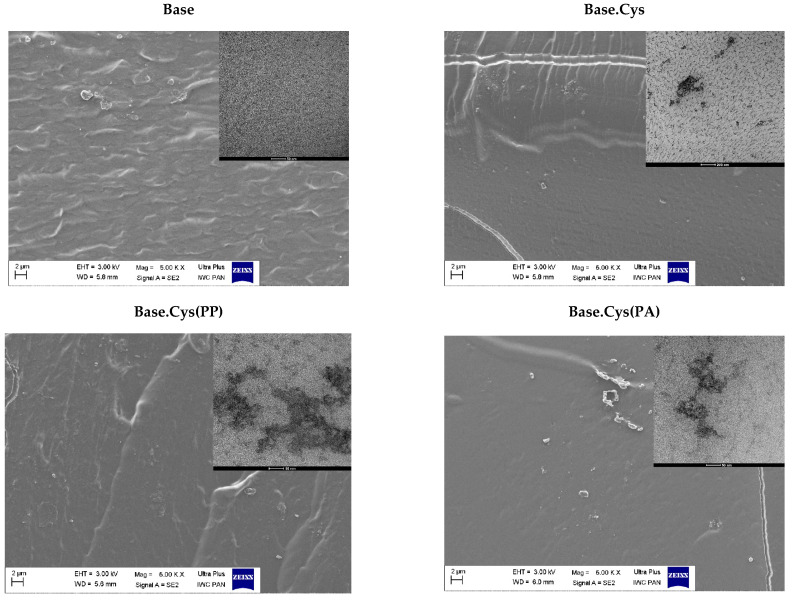
SEM and TEM images for Base, Base.Cys, Base.Cys(PP), and Base.Cys(PA).

The SEM microscopic images of selected gelatin matrices, such as Base, Base.Cys, Base.Cys(PP), and Base.Cys(PA), were obtained at a 5000×magnification (Figure 7). Material breakthroughs containing the cysteine introduced alone or preceded by the addition of the PP and PA initiators were imaged by scanning electron microscopy (SEM), and they are shown in Figure 7.

In the topography images of the breakthroughs of the Base material, a heterogeneous, undulating surface was observed, which could be attributed to the presence of suspended PVA particles. In the Base.Cys sample, a more ordered structure with local highlights and undulations resembling waves is visible. Areas with a homogeneous, smooth surface are also present.

The Base.Cys(PP) and Base.Cys(PA) samples showed partially homogeneous and locally irregular structures, with more noticeable heterogeneity evident in the Base.Cys(PP) sample. Images of this sample revealed a very rough surface with prominent hump-like highlights or fuzzy foliations. In the case of the Base.Cys(PA) sample, the presence of fine particles with diameters exceeding 2 μm was observed, which may represent residual PVA or unreacted initiator particles. These particles form single agglomerates or clusters visible at 50,000× magnification.

The results obtained by SEM are consistent with those obtained by transmission electron microscopy (TEM). The TEM observations confirmed the presence of numerous precipitates, corresponding to the undulating structure observed in the SEM images at lower magnifications.

The TEM studies by electron microscopy demonstrate a homogeneous distribution of components in a gelatin matrix containing protein hydrolysate and PVA. The introduction of cysteine as a crosslinking agent contributed to the formation of irregular clusters and the dispersion of the amino acid in the gel structure, creating a characteristic mosaic arrangement of components.

The use of potassium persulfate (PP) as an initiator resulted in a more uniform distribution of cysteine in the Base polymer matrix. Chemical reactions initiated by PP lead to the generation of free radicals in a predictable and controlled manner, which promotes the formation of stable crosslinks and a more homogeneous network structure. When phthalic anhydride (PA) was used, the gel structure was less uniform, allowing the formation of irregular cysteine clusters in the polymer matrix.

### 2.9. SEC Analysis

**Figure 8:**
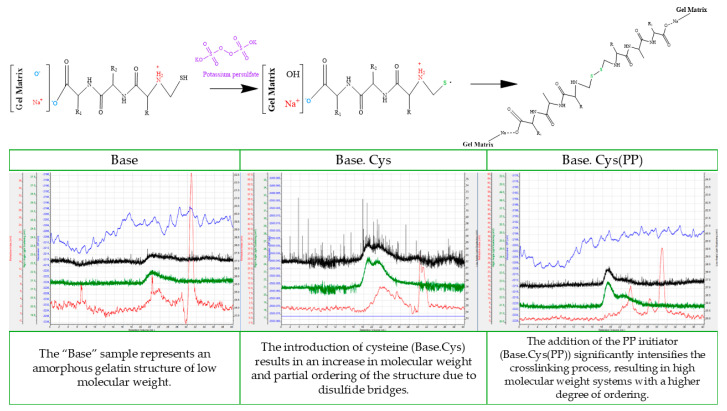
Probable mechanism of PP crosslinking of a fragment of a gelatin matrix macromolecule terminated with the amino acid cysteine and chromatograms for Base, Base.Cys., and Base.Cys(PP).

SEC analysis with triple detection was performed for the selected materials—Base, Base.Cys, and Base.Cys(PP). Polymer solutions of 5 mg/mL were prepared and filtered through syringe filters with a pore diameter of 0.2 μm before entering the column. The samples dissolved in the solution for 20 h and passed easily through the strainers. After the solutions were prepared, they were introduced into a GPC/SEC chromatography column, and the resulting chromatograms of the samples are shown in the corresponding figures. The recorded signals showed varying intensities for the following detectors: Refractive Index (RI), Right Angle Light Scattering (RALS), and Low Angle Light Scattering (LALS). There were no significant signals from the viscometric detector, as shown in Figure 8.

The Base material, shown in Figure 8, was characterized by the presence of very low-intensity RI, RALS, and LALS signals, which were additionally highly noisy, especially in the case of RALS and LALS. Based on the extent of the signals and their limits, the approximate molar mass values of the polymers were estimated using linear calibration for dextrans and glucans. In all chromatograms, a clear signal was recorded at around 30.9 mL, probably originating from residual solvent or water in the material. In the RI detector, broad signals were recorded between 21.5 and 26.0 mL, with a maximum intensity between 23.7 and 24.1 mL. In the case of the RALS detector, signals were observed in the range from 20.7 to 25.0 mL, with a maximum intensity at around 22.5 mL. The molar mass values assigned to these signals, according to the calibration for glucan, ranged from 37,700 to 3400 g/mol for RI and from 57,900 to 5700 g/mol for RALS. For calibration to dextran, the values ranged from 29,600 to 2500 g/mol for RI and from 50,000 to 5400 g/mol for RALS, respectively. The maximum molar masses for RI signals were estimated at 9300–11,500 g/mol when calibrated to glucan and 8900–10,700 g/mol when calibrated to dextran, while for the RALS signals, the values were 22,000 g/mol when calibrated to glucan and 18,000 g/mol when calibrated to dextran.

In the case of the Base.Cys material, shown in Figure 8, the RI signals were clear, with a maximum at an elution volume of 22.3 mL. In all chromatograms, an intense signal was noted at around 30.5 mL, which was attributed to residual solvent or water. Based on the linear calibration for dextrans and glucans, the molar mass values of the analyzed polymers were estimated to be about 24,500 g/mol for the calibration for glucan and 19,700 g/mol for the calibration for dextran. The RALS detector showed a bimodal character of the signal with maxima at values of 19.2 and 21.1 mL, indicating the presence of two fractions with different molar masses.

A similar bimodal nature of the RALS signal was noted for the Base.Cys(PP) material, shown in Figure 8. The range of molar masses for RI was similar to the values obtained for the earlier samples. Due to the bimodality of the RALS signal, molar masses were estimated to range from 44,300 to 5700 g/mol for calibration to glucan and from 35,400 to 5400 g/mol for calibration to dextran, with an elution volume range from 21.2 to 25.0 mL. In the absence of an RI signal, a visible RALS signal may indicate the presence of high molar mass products, whose concentration, however, is close to zero. In the case of the Base.Cys(PP) sample, no molar mass was assigned to the signal in the range of 17.9 to 21.2 mL. However, based on the bimodality of the RALS signal in this range, molar masses were estimated to range from 260,900 to 44,300 g/mol for the calibration to glucan and from 185,000 to 35,400 g/mol for the calibration to dextran. The second range of elution volumes from 21.2 to 25.0 mL was assigned molar masses of 44,300 to 5700 g/mol (calibration to glucan) and 35,400 to 5400 g/mol (calibration to dextran), respectively.

Based on the analysis, it was found that crosslinking with amino acids, especially cysteine (Base.Cys), and their combination with initiators (Base.Cys(PP)) increases the values of molecular weights compared to the native system. The average molar mass values increase from the range of 22–18 kDa to 24.5–19.7 kDa, depending on the calibration used for glucan or dextran. These results confirm the formation of new structures and the occurrence of interactions in gelatin matrices of type A collagen, crosslinked according to the designed methods [37,38].

The process of crosslinking with amino acids, especially cysteine, in combination with the PP initiator (Figure 8), contributes to an increase in the molar mass and the stabilization of the matrix structure, which is reflected in the more intense and clear signals of the RI and RALS detectors. The bimodal nature of the signals in the Base.Cys and Base.Cys(PP) samples indicates the existence of different molecular fractions, which can be attributed to crosslinking inhomogeneities. The presence of signals corresponding to large molar masses in the range of low chromatographic elution volumes suggests the formation of highly crosslinked structures with large molecular masses, albeit occurring at low concentrations.

The increase in molar masses in Base.Cys and Base.Cys(PP) samples indicates the efficiency of the crosslinking process and the structural stabilization of the material in the presence of the PP initiator. The results suggest that a properly selected initiator can effectively increase the molar mass and structural complexity of the material, which is a key element in the design of functional biopolymer materials.

### 2.10. TOF SIMS Analysis

**Figure 9:**
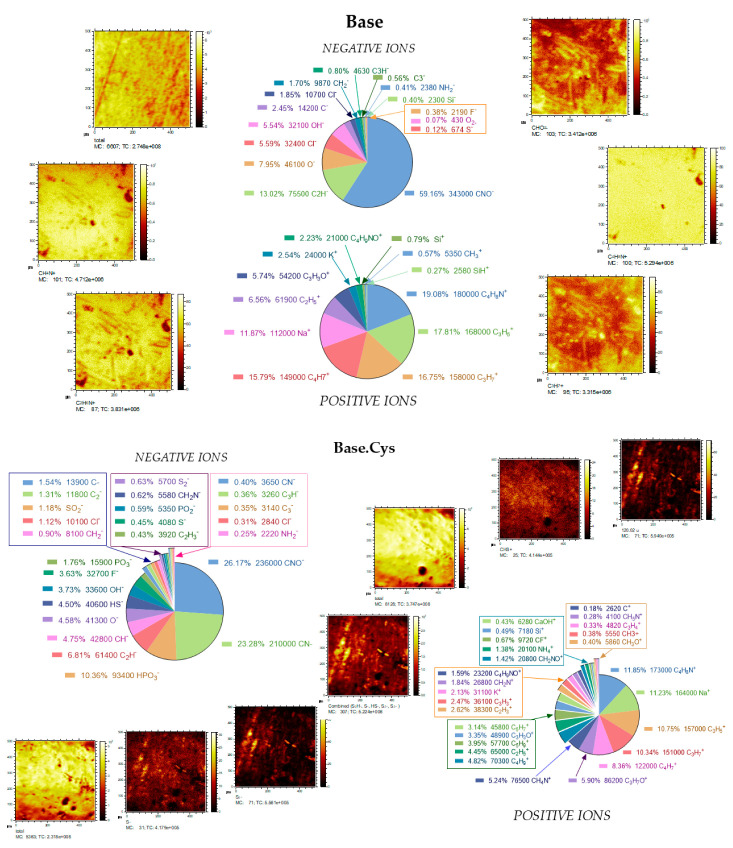
Analysis of surface ionic composition of the Base and Base.Cys samples using the TOF-SIMS technique—comparison of positive and negative ion distribution.

The results of the TOF-SIMS analysis for the Base and Base.Cys samples provide important information about the chemical composition, ion distribution, and structural changes due to the introduction of cysteine. The analysis of ion maps for positive and negative ions reveals clear differences between the studied samples, which allows the precise characterization of their composition and potential crosslinking processes, as shown in Figure 9.

For the Base sample, positive ion analysis revealed the dominance of the CNO^+^ fragment (59.16%), suggesting the presence of nitrogen-containing organic structures. The significant proportion of organic ions, such as C_2_H_5_^+^ (6.56%), C_3_H_7_^+^ (15.79%), and C_3_H_3_^+^ (16.75%), confirms the presence of simple hydrocarbons and their derivatives in the matrix. In addition, the presence of Na^+^ ions (11.87%) and fragments of Si^+^ (0.79%), SiH^+^ (0.27%), and SiOH^+^ indicates the possible contribution of salts, as well as silica impurities, which may come from the synthesis process or the environment.

For the negative ion maps of the Base sample, the most abundant fragments are CNO- (59.16%) and C_2_H- (13.02%), which clearly confirms the organic nature of the sample. The presence of OH- (5.54%) and O- (7.95%) suggests a significant content of oxygen compounds, probably in the form of hydroxyl groups or water. In contrast, the low proportion of sulfur ions, such as S- (0.12%), indicates the absence of a significant number of sulfides in the matrix.

After the introduction of cysteine into the matrix, significant changes in the ionic composition were observed in the Base.Cys sample, which clearly confirms the contribution of this amino acid to the modification of the sample. In the positive ion maps, the proportion of organic fragments such as C_2_H_3_^+^ (10.34%), C_2_H_5_^+^ (11.23%), C_3_H_7_^+^ (10.75%), C_4_H_7_^+^ (4.82%), and C_4_H_9_^+^ (5.24%) increased. The presence of more complex hydrocarbon fragments indicates changes in the organic structure of the sample have occurred, probably due to the participation of cysteine in the crosslinking processes. The presence of C_4_H_4_N^+^ (11.85%) and C_3_H_6_O^+^ (5.90%) fragments suggest the development of nitrogen- and oxygen-containing structures, which is consistent with the expected effects of amino acid introduction. TOF SIMS analyses confirmed the presence of a 120.02-unit quasimolecular ion consisting of a molecule and a weakly bound CSH^+^ positive ion. The fragment may originate from the cysteine structure interacting with the structure through S-C, S-S, or SH covalent bonds. The detection of S-S bonds further supports the formation of disulfide bridges, consistent with the chemical nature of cysteine and its ability to form covalent disulfide bonds under oxidative conditions [39]. The polymeric structure and degree of crosslinking of the systems are also affected by fragments derived from the negative phosphorus or nitrogen ions found in the gelatin matrix—a glycoproteid derivative of casein—used to create the matrix.

Negative ion maps for the Base.Cys sample reveal a marked increase in the proportion of sulfur ions, such as HS- (4.50%) and S^2−^ (0.63%), confirming the presence of disulfide bridges, characteristic of cysteine-mediated crosslinking. There was also a significant increase in CN- (23.28%) and CNO- (26.17%), which indicates a higher concentration of nitrogen-containing compounds. On the other hand, the increased content of phosphates, such as HPO_3_- (10.36%) and PO_3_- (1.76%), may be due to the interaction of the gelatin matrix with cysteine or the influence of other components present in the sample.

The results for the Base and Base.Cys samples clearly indicate significant differences due to the introduction of cysteine. The presence of sulfur and nitrogen fragments in the Base.Cys sample indicates crosslinking through disulfide bridges and enrichment of the matrix with more complex organic structures. The introduction of cysteine also induced significant changes in phosphate composition and hydrocarbon distribution, as evidenced by the changes in the positive and negative ion maps. The introduction of cysteine into the biopolymer matrix leads to an increase in the chemical and mechanical stability of the material. The increase in the proportion of nitrogen- and sulfur-containing fragments clearly indicates the active participation of cysteine in the crosslinking process. At the same time, changes in the ionic composition and distribution of phosphate fragments may be due to reactions between the functional groups of proteins and cysteine, further enhancing the matrix structure.

The TOF-SIMS results clearly confirm the effect of cysteine on the chemical and structural modification of the sample. The presence of disulfide bridges and changes in the composition of organic fragments indicate the effective introduction of crosslinking, which is crucial for improving the properties of biopolymer materials.

The crosslinking agent affects the chemical structure of the produced gel systems. Changes in the ionic composition of the TOF-SIMS sample (e.g., the appearance of CSH^+^ ions or organic fragments such as C_4_H_4_N^+^) confirm the chemical modifications caused by the introduction of cysteine and the initiators. The presence of disulfide bridges (S-S) and nitrogen and oxygen fragments indicates the specific role of the crosslinking agent in shaping the polymer structure. The increased molecular weights of GPC/SEC and the low elution volumes in Base.Cys and Base.Cys(PP) gels indicate a higher degree of crosslinking, which reflects the effectiveness of the crosslinking agents in stabilizing the structure. Based on thermal studies and mechanical analyses, the enhanced hardness of the gels crosslinked with cysteine and initiators such as PA and PP is observed, as well as a higher thermal stability (confirmed by TGA and XRD), confirming that crosslinking agents significantly affect the chemical structure and physicochemical properties. The surface hydrophobicity and polarity studied by contact angle measurements showed that the introduction of initiators changes the surface character from more hydrophobic to polar. This phenomenon results from the presence of new functional groups introduced by the crosslinking agents.

### 2.11. Microbiological Studies

The microbiological studies were based on the determination of the fungistatic effect using the filamentous fungus Aspergillus brasiliensis via the B method, and studies involving the bacteria Pseudomonas aeruginosa via the C method. Details about the sample preparation and the characteristics of the mycelium and bacterial cultures used are included in the methodology section. The materials chosen for the study were Base and Base.Cys.

In the determination of the fungistatic effect using method B, the materials tested were treated with a suspension of fungal spores in the presence of a full-strength medium providing a carbon source for the fungi. Three batches of samples were prepared, five in each batch. Batch 0 consisted of the control samples stored at a room temperature of about 24 °C. Batch I consisted of samples inoculated with the Aspergillus brasiliensis fungus and incubated. Batch S included sterile samples stored under the same conditions as batch I samples, which were additionally exposed to UV light in a laminar chamber for 20 min.

A total of 0.1 mL of the prepared spore suspension was pipetted onto the surface of each batch I sample and the agar surface. Batch S samples were only exposed to UV light for 20 min to avoid contact with the germicidal solution, i.e., 70% ethanol solution, which caused the test samples to dissolve. No visual changes were observed after the irradiation of the batch S samples.

**Figure 10:**
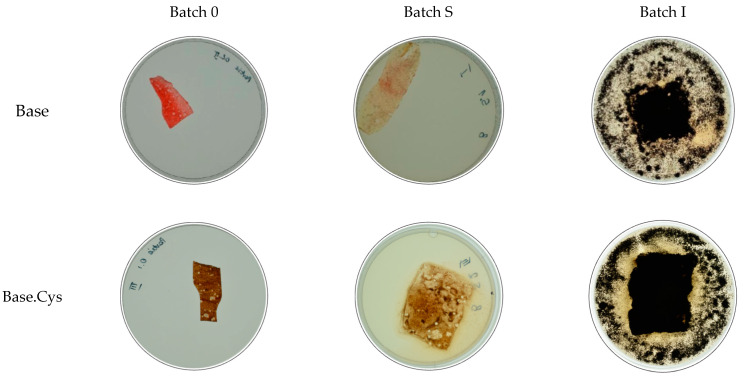
Microbiological tests for Base and Base.Cys performed using method B.

Batch I and S samples were incubated for four weeks at 24°C ± 1 °C. During the incubation, it was found that all test samples swelled and significantly increased their dimensions. The images (Figure 10) show the appearance of the samples from batch 0, batch I, and batch S for the different materials tested.

Upon completing the 4-week incubation period, the samples were visually evaluated for the growth of filamentous fungi on the samples. The evaluation was conducted according to the scale. Table 4 summarizes the results of the visual evaluation of the samples for the tested materials—Base and Base.Cys.

In the experiment conducted for all the materials tested, the intensity of microbial growth was found to be 5, indicating the absence of a fungistatic effect. The materials were quickly decomposed under the influence of microorganisms, making them excellent candidates for sustainability-based applications. Their biodegradability makes them environmentally safe and supports the natural cycles of matter in microbial ecosystems.

**Figure 11:**
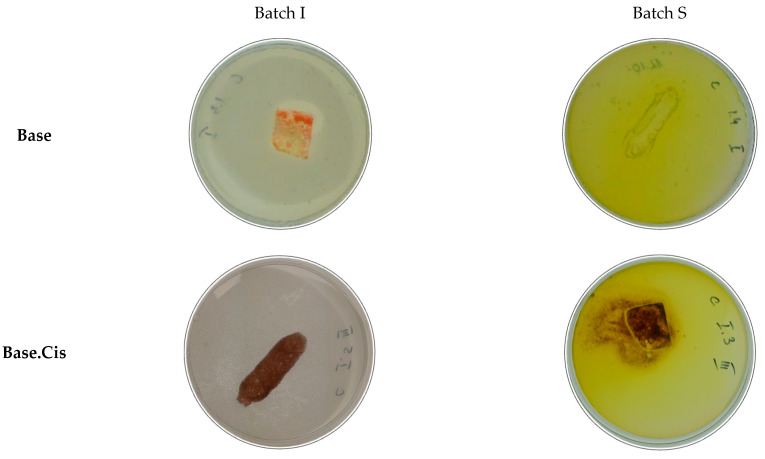
Microbiological tests for Base and Base.Cys performed using method C.

In the C-method study, the effects of Pseudomonas aeruginosa bacteria on Base and Base.Cys test materials were evaluated using an incomplete medium, i.e., agar with mineral salts. Three batches of samples were prepared (Figure 11), five in each batch. Batch 0 consisted of control samples, stored under room conditions at approximately 24 °C. Batch I consisted of samples inoculated for incubation. In its preparation, inoculated agar was used, which was poured into Petri dishes, and after clotting, individual samples were applied to its surface, which was then reflooded with inoculated agar.

Batch S consisted of sterile samples, stored under the same conditions as batch I samples. Batch S samples were exposed to UV light in a laminar chamber for 20 min. For their preparation, unclotted agar with mineral salts was used and poured onto Petri dishes. After clotting, individual samples were applied to the surface of the agar and then poured back onto the uninoculated agar. The plates were exposed to UV light for 20 min to sterilize them.

The samples were incubated for four weeks at 29 °C ± 1 °C. After incubation, microbial growth was observed on the surface of the samples and in their immediate surroundings, indicating the presence of nutrients that support microbial growth (Figure 11). Pseudomonas aeruginosa, as an organism capable of utilizing a variety of carbon and nitrogen sources, found a suitable substrate for growth in the tested materials. The lack of antimicrobial activity suggests that the tested materials could be applied in composting systems, biodegradable packaging, or the production of environmentally friendly biomaterials.

The presence of microorganisms on the surface and around the samples indicates that the material provides nutrients and shows biodegradability. This demonstrates its potential for use in environments that require high levels of biodegradability and ecological compatibility, which complies with sustainability goals.

## 3. Materials and Methods

### 3.1. Materials

The following raw materials were used to prepare the polymer matrix: gelatin with a Bloom index of 200° (pH 5–8, Mw 30 kDa, FoodCare Sp. Z.o.o., Zabierzów, Poland); glycerin (density 1.26 g/cm^3^, Mw 92 kDa, pH 6–8, flash point 160 °C, CHEMPUR, Piekary Śląskie, Poland); sodium hydroxide (pH approx. 13–14, solidification point 12 °C, viscosity 22 mPas at 40 °C, EUROCHEM BGD Sp. Z.o.o., Tarnów, Poland); polyvinyl alcohol (PVA, mol. wt. 72,000 g/mol, POCH S.A., Gliwice, Poland); keratin hydrolysate (enzymatic hydrolysate produced according to patent procedure PL214486); L-cysteine (HSCH_2_CH(NH_2_)CO_2_H, (R)-2-amino-3-mercaptopropionic acid, mol. wt. 121.2 g/mol, CHEMPUR, Piekary Śląskie, Poland); trans-4-hydroxy-L-proline (C_5_H_9_NO_3_, (2S,4R)-4-hydroxypyrrolidine-2-carboxylic acid, mol. wt. 131.13 g/mol, Sigma-Aldrich Chemie GmbH, Steinheim, Germany); DL-proline (C_5_H_9_NO_2_, (±)-pyrrolidine-2-carboxylic acid, mol. wt. 115.13 g/mol, Sigma-Aldrich Chemie GmbH, Steinheim, Germany); L-histidine (C_6_H_9_N_3_O_2_, (S)-2-amino-3-(4-imidazolyl)propionic acid, mol. wt. 155.15 g/mol, Sigma-Aldrich Chemie GmbH, Steinheim, Germany); potassium persulfate (PP, K_2_S_2_O_8_, mol. wt. 270.32 g/mol, CHEMPUR, Piekary Śląskie, Poland); phthalic anhydride (PA, C_8_H_4_O_3_, mol. wt. 148.12 g/mol, Sigma-Aldrich Chemie GmbH, Steinheim, Germany); and azobis(isobutyronitrile) (AIBN, 2,2′-azobis(2-methylpropionitrile), 98%, (CH_3_)_2_C(CN)N=NC(CH_3_)_2_CN, mol. wt. 164.21 g/mol, Sigma-Aldrich Chemie GmbH, Steinheim, Germany). The following natural dyes were used to color the compositions: quinoline yellow (E104, 95%) and cochineal red (E124, purum pur., 1-(1-naphthylazo)-2-hydroxy-naphthalene-4′,6,8-trisodium trisulfonate, Molecu SYNCHRO, Łódź, Poland).

### 3.2. Sample Preperation

To fabricate the polymer gel, a reactor equipped with a temperature control system and a mixing mechanism was used. Gelatin-based compositions were crosslinked using two methods. In the first method, amino acids were used as crosslinking agents, while in the second method, the action of amino acids was further enhanced with ionic and redox initiators. The reactor was placed in an oil bath to ensure uniform heat distribution. The reactor was loaded with gelatin (Bloom index 200°) at a concentration of 70 PBW, followed by the addition of measured amounts of polyvinyl alcohol (PVA) with a molecular weight of 72,000 g/mol at a concentration of 5 PBW, and enzymatically hydrolyzed keratin at the same concentration. Glycerin was added to the system at a concentration of 18 PBW, along with an appropriate dye at 0.01 PBW by weight to facilitate differentiation and identification of the prepared compositions. To regulate pH regulation, initiate crosslinking reactions, and dissolve components such as protein hydrolysate, 1 PBW NaOH was added.

In the first method, the following four amino acids in aqueous solutions at a concentration of 0.5 PBW were used as crosslinking agents: cysteine (Base.Cys), hydroxyproline (Base.Hyp), proline (Base.Pro), and histidine (Base.His). In the second method, before adding cysteine, the following crosslinking initiators were introduced into the system: potassium persulfate (PP), phthalic anhydride (PA), and azobisisobutyronitrile (AIBN), each at a concentration of 2.25 PBW. The adopted procedure enabled the production of polymer gel via a solvent-casting method.

The resulting gels were subjected to thermostabilization under a pressure of approximately 100 bar at 100 °C, resulting in a stable polymeric material suitable for further analysis. All of the ingredients and their respective amounts in the compositions are presented in Table 5.

### 3.3. Methods

#### 3.3.1. Statistical-Mechanical Analysis

Dynamic mechanical strength tests were performed to determine the tensile strength of the obtained gel compositions. Samples in the shape of standard “paddles” were prepared according to PN-EN ISO 3167:2014-09 [40], using a Zwick ZCP020 die cutter (ZwickRoell GmbH & Co. KG, Radeberg, Germany). At least five samples were tested to minimize the standard error. The tests were conducted according to the PN-ISO 37:1998 [41] standard using a Zwick 1422 testing machine (ZwickRoell GmbH & Co. KG, Radeberg, Germany).

During the tests, the samples were stretched to failure at a stretching speed of 50 mm/min and an initial force of 0.1 N. The device recorded the force required to stretch the sample, expressed in newtons [N], and then converted it to tensile stress (TS), expressed in megapascals [MPa]. Additionally, the elongation at break (Eb) was recorded as a percentage value [%].

To determine the static mechanical properties of the materials, the hardness of the gel compositions was also measured. The Shore hardness test was conducted using a Shore durometer type “A” (type 3130/3131, ZwickRoell GmbH & Co. KG, Ulm, Germany), in accordance with ISO 7619-1 [42]. The test applied a pressing force of 12.5 N. The procedure was repeated five times at different locations on each sample and the final result was reported as the mean value of the readings, including the standard error.

#### 3.3.2. Fourier Transform Infrared Spectroscopy

A Nicolet 6700 spectrophotometer (Thermo Scientific, Waltham, MA, USA) was used to characterize the structure and functional group interactions in the macromolecular segments of the gel materials. Measurements were carried out in the FTIR wavelength range of 4000–400 cm^−1^ with a resolution of 0.25 cm^−1^.

#### 3.3.3. GPC/SEC Analysis

The chromatographic analysis (GPC/SEC) utilized the selected additive and involved preparing materials in an aqueous 0.1% NaN_3_ solution. After three days, the polymer materials almost completely dissolved. The chromatography conditions were as follows: HPLC-501 K (Knauer, Berlin, Germany) from an RI detector (LDC, Riviera Beach, FL, USA) and a dual-angle light scattering detector (T60A; Viscotek, Houston, TX, USA). The gel permeation chromatography system was characterized by three TSK-GEL columns (G5000 PWXL, G3000 PWXL, G2500 PWXL; 7.8 × 300 mm; Tosoh Bioscience, Tokyo, Japan) at a temperature of 26 °C. The analysis was conducted using OmniSEC Software (version 11.32; Malvern Panalytical, Malvern, UK). The eluent used was a 0.1% NaN_3_ solution, with a flow rate of 1.0 mL min^−^^1^. Detector designations included RI (Refractive Index detector), LS (Light Scattering detector), and Visc (Differential Viscometer).

The chromatographic analysis GPC/SEC used the selected additive and consisted of the preparation of materials in aqueous 0.1% NaN_3_. After 3 days, the polymer materials almost completely dissolved in the solution. The conditions of the chromatography are as follows: Knauer Pump (HPLC -501 K, Knauer, Berlin, Germany) from RI detector (LDC) and double Vasotec detector (T60A; scattering of light (RALLS and LALS) and differential viscometer). The characterization of the analysis system of gel exclusion chromatography (GPC/SEC) is as follows: Three columns TSK-GEL: G5000 PWXL, 3000 PWXL, 2500 PWXL (7.8 × 300 mm; Tosho); temp. 26 °C. Omni SEC Software. Eluent: 0.1% NaN3 solution; flow rate: 1.0 mL × mm^−1^. Detector designation: RI—refractometric detector; LS—scattering; Visc—differential viscometer.

#### 3.3.4. Surface Free Energy and the Degree of Crosslinking

The purpose of the goniometric study was to analyze the interaction of the solid substrate with the material wetting liquid and to find out the nature of the surface of the obtained gel compositions. The study was carried out using the Owens–Wendt method through the use of an OCA 15EC goniometer (Dataphysics, Filderstadt, Germany) and consisted of placing drops of three solvents, of different polarity, on the surface of the gel films and measuring the wetting angle. The solvents were water, diiodomethane, and ethylene glycol. On this basis, the surface free Energy γS and its components, polar γS and dispersive ${\gamma S}_{d}$. were determined.

$$\gamma S={\gamma S}_{p}+{\gamma S}_{d}$$

The purpose of the study was, among other things, to determine the wetting angle on a stationary substrate. For this purpose, the following three different liquids were applied with a micropipette: distilled water, ethylene glycol, and diiodomethane. Each liquid had a specific role as follows: distilled water had a high polar component, while diiodomethane was a dispersive liquid. Based on the wetting angle measurements, the surface free energy (SEP) (γ [mJ/m^2^]) was calculated, defined as the work required to form a new unit of surface area in the separation process of two phases in equilibrium. The surface tension (σ [mN/m]) is a value describing the force acting tangentially to a surface per unit of its length, resulting from the imbalance of intermolecular forces at the interface. Although the units of these quantities are the same ([mJ/m^2^] = [mN/m]), the surface tension is a vector and surface energy is a scalar. The relationship between the two is described by the following equation:

$$\sigma =\gamma +S\frac{d\gamma }{dS}$$

where S is the unit of surface area. For liquids, once a new surface is created, the atoms assume new equilibrium positions, hence $\frac{d\gamma }{dS}=0$, and the equation is simplified to σ=γ(dlaS≠0). Therefore, for liquids, the measurable quantity is surface tension, while for biopolymer films it is surface energy. The results of the measurements of the wetting angles (Θ [°]) and components are expressed as follows: polar $\left(\gamma_{s}^{P}\left[\frac{mN}{m}\right]\right)$, dispersion $\left(\gamma_{s}^{D}\left[\frac{mN}{m}\right]\right)$, and surface energy (γ). The materials are shown in Table 2.

#### 3.3.5. Thermal Properties

Thermogravimetry and differential scanning calorimetry were carried out to determine the thermal stability, identify compositional components, and estimate their quantitative content in the sample. Measurements were performed using a Mettler Toledo TGA/DSC thermogravimetric analyzer (Mettler Toledo, Greifensee, Switzerland) in the temperature range from 25 °C to 900 °C, at a constant heating rate of 10 °C/min. The standard deviations of the results were within T_g_ ± 20 °C. The analyses were performed in an inert nitrogen atmosphere.

#### 3.3.6. HPLC Analysis

HPLC was performed using reversed-phase chromatographic separation with UV detection by RAVIMD Sp. z o.o., Łajski, Poland. After the gel compositions were completely dissolved, their amino acid concentrations under the peaks of the chromatographic curves were determined in units [mg/g]. Samples for the HPLC analysis were prepared by completely dissolving the biopolymer gel in an appropriate solvent and then filtering the solution. After filtration, the sample was injected into the chromatograph, and the amino acid concentrations were determined from the areas under the chromatographic curves.

#### 3.3.7. Elemental Analysis

The determination of the percentage of CHN elements by elemental analysis using the UNICUBE analyzer (Elementar Analysensysteme GmbH, Berlin, Germany) was determined under contract by the Company Environmental Laboratory of Functional Materials and Nanotechnology of the Department of Catalysis and Organometallic Chemistry of the Faculty of Chemistry, Warsaw University of Technology.

#### 3.3.8. Softening Point

The softening temperature tests were carried out using a Vicat apparatus (D-Vicat.HDT/3/300FA, CEAST, Pianezza, Italy). The purpose of the measurement was to determine the softening temperature of the composition. The procedure consisted of gradually plunging a steel needle into a test sample of standard dimensions, with a constant load and increasing temperature. The sample was immersed in an oil bath with an initial temperature of 23 °C, and the bath was heated at a rate of 50 °C or 120 °C per hour until the needle penetrated to a depth of 1 mm. Samples tested by the Vicat method had to have a thickness from 3 to 6.5 mm and minimum width and length dimensions of at least 10 mm. Each measurement was repeated three times, and the average softening temperature value for the sample was calculated.

#### 3.3.9. XRD Analysis

An XRD diffractometer from Bruker D8 Advance (Bruker, Billerica, MA, USA) equipped with a Johansson monochromator (λCu Kα1 = 1.5406 Å) and a strip detector (LynxEye, Drottninggatan 95A, 113 60 Stockholm, Sweden) was used to determine the presence of a crystalline phase in the compositions studied. The angular range is 10–70 2*θ*, step 0.03.

#### 3.3.10. TOF SIMS Analysis

The distribution of ions on the sample surface was obtained using TOF-SIMS time-of-flight secondary ion mass spectrometry. Measurements were performed on a TOF-SIMS.5 spectrometer (ION-TOF GmbH, Berlin, Germany) operating in Bi^3+^ mode. The obtained samples were transferred without special pre-treatment to the analytical chamber. The base pressure in the chamber was below 2 ×10^−9^ mbar. Before analysis, a few spots on the sample surface with an area of more than 300 × 300 μm or 500 × 500 μm were selected. The mode of operation of the instrument was spectroscopy at 30 keV energy and Bi3+ ion current conditions of 0.5 pA. A low-energy electron gun and surface potential mode were used to reduce sample charging during the analysis. Internal mass calibration was carried out using the mass of several ions from CH_3_^+^ to C_4_H_8_NO^+^ ions in positive polarization and from C- to C_5_^−^ ions in negative polarization. The sputtered sample was etched with oxygen ions operating at an energy of 2 keV and etched over an area of 500 × 500 µm.

#### 3.3.11. Surface Morphology

A Zeiss Ultra Plus scanning electron microscope (SEM/EDS) (Bruker, Massachusetts, USA) was used to analyze the morphology of the composition. Observations of the structure were made using a Philips XL30 environmental scanning electron microscope (ESEM) at an accelerating voltage of 10 kV. Prior to analysis, the samples were coated with a layer of carbon using a Cressington 208 HR system to ensure adequate electrical conductivity.

Transmission electron microscopy (TEM) images were taken with a Talos F200x(FE) transmission electron microscope at an accelerating voltage of 200 kV. Thin layers of the samples were precisely cut with a scalpel from their top surface. Slices of the samples were then transferred to a TEM Cu 300 mesh, previously coated with a drop of water to improve adhesion. After drying at room temperature, the samples were additionally treated with oxygen-argon plasma for 3 min to increase the contrast and quality of the images obtained.

#### 3.3.12. Microbiological Studies

Microbiological tests were designed to evaluate the effect of microorganisms on polymeric materials and were carried out at the Jagiellonian Innovation Center in Krakow, Poland, according to PN-EN ISO 846 [43], methods B and C.

**Determination of the fungistatic effect (method B)**

The evaluation of the fungistatic effect involved a carefully designed procedure to assess the resistance of the test materials to fungal growth. For this purpose, a strain of filamentous fungi, Aspergillus brasiliensis (ATCC 16404), was selected due to its robustness and ability to thrive in various conditions. The test was conducted using two types of culture media, a complete agar medium and a mineral salt solution enriched with glucose, which provided an optimal environment for fungal proliferation.

The testing process began with the preparation of fungal spores. Spores were harvested using a mineral salt solution supplemented with glucose, ensuring their viability and consistency. Following the guidelines specified in ISO 846, the spore suspension was carefully washed and centrifuged to remove impurities. This process resulted in a suspension with a density of approximately 10^6^ spores per milliliter, ready for application to the test materials.

To confirm the viability of the spores, a small volume of the prepared suspension was inoculated onto a full-nutrient medium. After an incubation period, the presence of abundant mycelial growth validated the effectiveness of the spore preparation, ensuring its suitability for the main experiment.

The prepared samples were then subjected to incubation under controlled conditions. The environment was maintained at a temperature of 24 °C ± 1 °C with a relative humidity exceeding 95%. The incubation period lasted for four weeks, during which the samples were closely monitored. The absence or presence of fungal growth on both the test materials and the surrounding medium was used as an indicator of fungistatic activity.

After the incubation period, each sample was thoroughly examined for evidence of fungal growth. Observations were documented using a standardized five-point scale as described in ISO 846. This assessment provided a clear representation of the intensity of fungal growth and any morphological changes that occurred in the test materials. Through this detailed evaluation, the fungistatic properties of the materials were effectively characterized

**Fungistatic Effect Assessment (Method C)**

The interaction between *Pseudomonas aeruginosa* and the tested material was evaluated using a nutrient-deficient agar medium with mineral salts (Table 6). The absence of bacterial growth on the agar around the sample indicated the material’s lack of nutrient content.

The bacterial cultures were grown on peptone agar with soybean casein and incubated for 24 h at 29 °C. A bacterial suspension was prepared in a sterile buffer solution with a density of approximately 10^6^ cells/mL. To confirm the bacterial viability, three drops of the suspension were added to 10 mL of sterile peptone agar with soybean casein. After a 48-h incubation at 29 °C, normal microbial growth was observed, confirming cell viability.

Subsequently, the prepared bacterial suspension (10^6^ cells/mL) was inoculated into molten mineral salt agar to achieve a concentration of approximately 50,000 cells per milliliter of agar, ensuring suitable conditions for further microbiological tests.

During the study, all tested materials dissolved when exposed to a 70% ethanol solution used as a bactericidal agent. Consequently, samples were dried at 45 °C for 4 h after immersion. However, partial melting of the samples was observed, counteracting the continuation of the tests in this form.

To overcome this limitation, samples from batch S were sterilized using UV radiation for 20 min instead of ethanol immersion. Post-irradiation, no visible changes in the appearance of the samples were observed, confirming the efficacy of the alternative sterilization method without compromising material integrity. Table 7 shows the assessment of mycelial growth based on growth intensity and its respective descriptions.

## 4. Conclusions

The introduction of scleroproteins such as collagen and the appropriate initiators into biopolymer matrices has enabled the development of materials with significantly improved mechanical, thermal, and biodegradable properties. As a result, such materials become promising candidates for applications in sustainable technologies and modern biomaterials. Among the tested amino acids, cysteine stood out as the most effective crosslinking agent, which was confirmed by the high hardness values of gels crosslinked with this amino acid compared to samples modified with other amino acids such as proline or histidine.

The results of the TOF-SIMS analyses revealed that the presence of cysteine in the biopolymer matrix leads to the formation of specific changes in the chemical structure, which is manifested by the presence of disulfide bridges (S-S), thiol interactions (S-H), and the appearance of quasimolecular ions such as CSH^+^. These structural modifications are crucial for improving the mechanical properties and stability of the material. Moreover, the use of initiators such as PA and PP allowed for a further increase in the hardness of the gel matrix by about 7% compared to the reference material, which indicates their efficiency in the crosslinking process.

Chromatographic analyses (GPC/SEC) indicated a significant increase in molecular weights in the Base.Cys and Base.Cys(PP) samples, which indicates a high degree of crosslinking and structural stabilization. The presence of high molecular weights at low elution volumes confirmed the formation of strongly crosslinked structures. The results of thermal studies, such as TGA and XRD, showed that cysteine-crosslinked gels are characterized by higher thermal stability compared to gels modified with proline or histidine, which makes them particularly attractive in applications requiring resistance to high temperatures.

Further contact angle studies revealed differences in surface hydrophobicity between gels crosslinked only with amino acids and those with the addition of initiators. The gels with initiators showed a greater number of polar interactions, which reduced their hydrophobicity, but at the same time increased their compatibility with water, which may be beneficial in some biomedical applications.

Observations on the growth of microorganisms on the gel surface confirmed its biodegradability, indicating the material’s compliance with the principles of sustainable development. Thanks to these properties, cysteine-crosslinked biopolymer gels can be used in environments requiring ecological compatibility. Finally, the TOF-SIMS spectral analysis and the mechanical, thermal, and structural properties of the gels clearly confirm the key role of crosslinkers in shaping the properties of the final material.

## Figures and Tables

**Table 1:** Shore A hardness measurements with standard deviation.

Sample	H ± dH [°Sh]	TS_b_ [MPa]	E_b_ [%]
Base	77.4 ± 0.3	2.1 ± 0.6	1.8 ± 0.1
Base.Cys	71 ± 3	11 ± 1	4.4 ± 0.2
Base.Hyp	64 ± 10	9.4 ± 0.2	7 ± 1
Base.Pro	65 ± 3	6.3 ± 0.4	3.4 ± 0.1
Base.His	64 ± 1	7.9 ± 0.4	4.6 ± 0.2
Base.Cys(PP)	84 ± 3	10.4 ± 0.1	7.73 ± 0.08
Base.Cys(PA)	46.7 ± 0.7	6.1 ± 0.1	2.63 ± 0.02
Base.Cys(AIBN)	81 ± 2	4.16 ± 0.01	1.24 ± 0.03

Legend: TS_b_—tensile strength [MPa]; E_b_—relative elongation at break [%].

**Table 2:** Wavenumbers for the following samples: Base; Base.His; Base.Hyp; Base.Pro; Base.Cys; Base.Cys(PP); Base.Cys(PA); and Base.Cys(AIBN).

Sample	Base	Base.His	Base.Hyp	Base.Pro	Base.Cys	Base.Cys(PP)	Base.Cys(PA)	Base.Cys(AIBN)	
Wavenumber (cm^−1^)			850		849	849	853	848	Out-of-plane bending vibrations, C-H, C-O, C-N)
		922	921	922	921	921	922	922	
	1035	1038	1035	1035	1036	1034	1035	1035	Stretching vibrations, C-O and C-N
					1108				
	1239	1238	1238	1238	1238	1239	1239	1237	Stretching vibrations, C-N; bending vibrations, N-H
	1334	1333	1335	1331		1334	1335	1336	Deformation vibrations, C-H; stretching vibrations, CH_2_ and CH_3_
	1405	1405	1404	1404	1398			1404	Deformation vibrations, CH_3_; asymmetric COO^−^ vibrations
	1449	1448	1448	1448	1451	1449	1448	1448	Deformation vibrations, CH_2_ and CH_3_
	1536	1534	1544	1537	1537	1537	1532	1540	Amide II, N-H bending and C-N stretching
	1629	1629	1633	1629	1632	1632	1632	1633	Amide I, C=O and C-N stretching
		2933	2935	2936	2935	2934	2935	2933	Asymmetric stretching vibrations, CH_3_ and CH
	3277	3282	3282	3278	3279	3281	3280	3281	N-H and O-H stretching vibrations

**Table 3:** Comparison of polar and dispersion components and surface energies of samples synthesized with the addition of 0.5 M sodium hydroxide.

Composition	Θ _water_ [°]	Θ _ethyl glycol_ [°]	Θ _diiodomethane_ [°]	*γ*_*sP*_ [*m*N/*m*]	*γ*_*s*D_ [*m*N/*m*]	γ [mJ/m^2^]
Base	107.00 ± 3.8	53.3 ± 4.7	42.6 ± 5.5	2.8 ± 0.5	56.3 ± 8.8	59.1 ± 9.2
Base.Cys	124.8 ± 3.1	36.7 ± 3.5	38.8 ± 4.1	3.9 ± 1.2	76.1 ± 5.0	80.0 ± 5.2
Base.Hyp	123.3 ± 6.3	68.3 ± 3.4	52.8 ± 3.9	0.1 ± 0.0	36.2 ± 2.6	36.2 ± 2.6
Base.Pro	114.2 ± 1.4	62.6 ± 0.5	69.4 ± 4.3	3.1 ± 0.8	59.9 ± 3.5	63.0 ± 3.6
BaseHis	103.6 ± 5.2	55.9 ± 5.2	50.9 ± 3.4	0.1 ± 0.0	34.4 ± 3.3	34.5 ± 3.3
Base.Cys(PP)	62.0 ± 0.9	75.4 ± 5.2	56.4 ± 2.7	19.3 ± 1.2	20.1 ± 1.3	39.3 ± 1.8
Base.Cys(PA)	42.4 ± 4.3	45.2 ± 1.3	54.2 ± 5.3	36.7 ± 5.5	8.1 ± 2.4	44.8 ± 6.0
Base.Cys(AIBN)	55.6 ± 4.1	61.2 ± 5.5	59.5 ± 3.3	18.5 ± 2.7	18.9 ± 1.7	37.4 ± 3.2

Legend Θ [°]—contact angle; *γ*_*sP*_ [*m*N/*m*]—polar component; *γ*_*s*D_ [*m*N/*m*]—dispersive component; γ [mJ/m^2^]—surface free energy SEP.

**Table 4:** Results of visual evaluation of filamentous fungal growth on tested plastics.

	Batch 0	Batch S	Batch I
Base	0	0	5
Base.Cys	0	0	5

**Table 5:** Components used in the synthesis of biopolymers. Amounts are presented in parts by weight [PBW].

Ingredients/PBW	Composition Name							
	Base	Base.Cys	Base.Hyp	Base.Pro	Base.His	Base. Cys(PP)	Base. Cys(PA)	Base. Cys(AIBN)
Gelatin [PBW]	70							
Glycerin [PBW]	18							
NaOH [PBW]	1							
PVA [PBW]	5							
Keratin hydrolysate [PBW]	5							
Cysteine [PBW]	-	0.5				0.5	0.5	0.5
Hydroxyproline [PBW]			0.5					
Proline [PBW]				0.5				
Histidine [PBW]					0.5			
Potassium persulfate [PBW]						2.25		
Phthalic anhydride [PBW]							2.25	
Azobis(isobutyronitrile) [PBW]								2.25
Water [PBW]	100							

**Table 6:** Fungistatic effect assessment by method C—technical data.

Test Parameter	Details
Culture media	Mineral salt agar: inoculated and non-inoculated
Microorganism strain	*Pseudomonas aeruginosa* ATCC 9027
Incubation conditions	29 °C ± 1 °C for 4 weeks

**Table 7:** Mycelial growth assessment.

Growth Intensity	Description
0	No visible growth under a microscope
1	Microscopic growth not visible to the naked eye
2	Visible growth covering up to 25% of the sample surface
3	Visible growth covering up to 50% of the sample surface
4	Significant growth covering more than 50% of the surface
5	Intense growth covering the entire surface

## Data Availability

Data are contained within the article.

## Supplementary Materials

The following supporting information can be downloaded at: https://www.mdpi.com/article/10.3390/molecules30030627/s1, Supplement S1: FTIR analysis.
