# Supplementary material for: High-Performance Structures of Biopolymer Gels Activated with Scleroprotein Crosslinkers
Source: Molecules. 2025 Jan 31;30(3):627. doi: 10.3390/molecules30030627 (PMC11820096; doi:10.3390/molecules30030627)
Supplement: Supplementary file 1 [file molecules-30-00627-s001.zip › molecules-3394935-supplementary.pdf]

Supplement S1. Table of FTIR spectra

Sample	Base	Base.His	Base.Hyp	Base.Pro	Base.Cys	Base.Cys(PP)	Base.Cys(PA)	Base.Cys(AIBN)	
Wavenumber (cm^{-1})			850		849	849	853	848	Out-of-plane bending vibrations, C-H, C-O, C-N)
		922	921	922	921	921	922	922	
	1035	1038	1035	1035	1036	1034	1035	1035	Stretching vibrations, C-O and C-N
					1108				
	1239	1238	1238	1238	1238	1239	1239	1237	Stretching vibrations, C-N; bending vibrations, N-H
	1334	1333	1335	1331		1334	1335	1336	Deformation vibrations, C-H; stretching vibrations, CH_2 and CH_3
	1405	1405	1404	1404	1398			1404	Deformation vibrations, CH_3; asymmetric COO^- vibrations
	1449	1448	1448	1448	1451	1449	1448	1448	Deformation vibrations, CH_2 and CH_3
	1536	1534	1544	1537	1537	1537	1532	1540	Amide II, N-H bending and C-N stretching
	1629	1629	1633	1629	1632	1632	1632	1633	Amide I, C=O and C-N stretching
		2933	2935	2936	2935	2934	2935	2933	Asymmetric stretching vibrations, CH_3 and CH
	3277	3282	3282	3278	3279	3281	3280	3281	N-H and O-H stretching vibrations

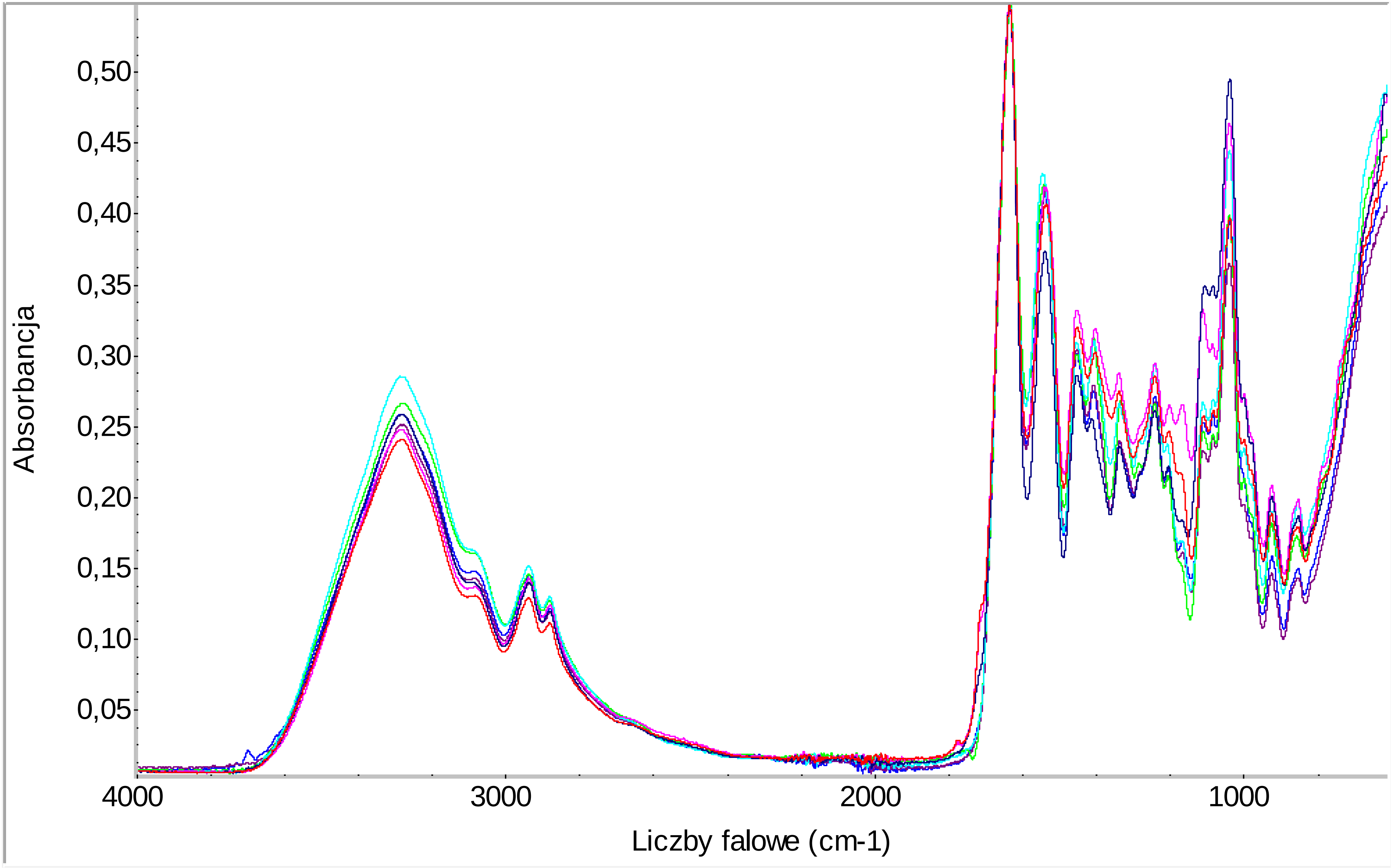

Base; Base.Pro; Base.Hyp; Base.Cys(AIBN) ; BaseHis; Base.Cys; Base.Cys(PP) ; Base.Cys(PA) ;
